# Which sucks more? Comparison of two novel direct-in-scope suction ureteroscopes to a conventional ureteroscope

**DOI:** 10.1007/s00345-025-05768-1

**Published:** 2025-08-17

**Authors:** Zhamshid Okhunov, Elizabeth A. Baldwin, Daniel Jhang, Ruben Crew, Ninous Betdashtoo, Kallan Richards, Grant Sajdak, Joshua Ghoulian, D. Duane Baldwin

**Affiliations:** Department of Urology, Loma Linda University Health, Loma Linda, CA USA

**Keywords:** Ureteroscopy, Direct in scope suction, Urolithiasis, Lithotripsy

## Abstract

**Introduction:**

: Novel Direct-In-Scope-Suction ureteroscopes (DISS-U) have recently been introduced to facilitate removal of small stone fragments. The purpose of this study was to compare two novel DISS-U to a conventional ureteroscope to determine efficiency for suctioning fluid and stone dust at different deflection angles.

**Methods:**

Three disposable ureteroscopes were evaluated, including two DISS-U with 5.1-Fr and a 3.6-Fr channels, and were compared to a conventional ureteroscope with 3.6-Fr channel. For DISS-U trials, a conventional suction system was set at maximal settings and tested at deflection of 0, 90, and 140°, to simulate upper, interpolar, and lower pole kidney stone evacuation under 3 conditions (empty channel, 2.2-Fr basket, and 0.038 guidewire). For the conventional ureteroscope, suctioning was performed manually using a 60-mL Luer lock syringe. Five trials were performed to measure the time in seconds to remove 1 gram of CaOx density BegoStones (0.25, 0.5, and 1 mm) and 50-mL of saline.

**Results:**

The 5.1-Fr DISS-U was the only scope that evacuated 0.5 mm stone fragments and evacuated them significantly faster from the upper and interpole compared to the lower pole (111.0 ± 27.7, 128.0 ± 29.1, and 377.7 ± 63.4 s at 0, 90, and 140°, respectively; *p* < 0.002). The 5.1-Fr DISS-U evacuated 1 g of dust faster than the 3.6 DISS-U and conventional ureteroscope at all angles (*p* < 0.007). The 5.1-Fr DISS-U and the 3.6-Fr DISS-U required less suction time for 50 mL of saline compared to the conventional ureteroscope at all angles (*p* < 0.001 for all). Using a 2.2-Fr basket increased suction time by 66.0% (*p* < 0.001) for the 3.6-Fr DISS-U and by 44% (*p* < 0.001) for the 5.1-Fr DISS-U, while the 0.038 guidewire increased suction time by 96% and 111%, respectively (*p* < 0.001). No ureteroscope was able to suction 1 mm stone fragments without clogging.

**Conclusions:**

The DISS ureteroscopes significantly outperformed the conventional ureteroscope in both suction speed and dust evacuation efficiency. The 5.1-Fr DISS-U demonstrated superior performance and is able to evacuate stone particles up to 0.5 mm.

## Introduction

Nephrolithiasis is a common urological condition affecting millions worldwide, with a prevalence ranging from 7 to 13% in North America that has only risen over the past few decades [1]. These stones can lead to significant morbidity, and the direct costs of diagnosing, treating, and preventing the recurrence of kidney stones are significant [2].

The treatment of kidney stones has evolved significantly with the advancement of endoscopic technology, offering minimally invasive alternatives to traditional surgical methods. According to the American Urologic Association Guidelines for surgical management of urolithiasis, retrograde intrarenal surgery (RIRS) is one of the recommended treatment options for stones less than 2 cm [3]. While shown to be effective in achieving a high stone free rate (SFR), removing large stone burdens with RIRS can be challenging, which can increase procedure time, and perioperative and postoperative morbidity [4]. During RIRS, residual stone dust, fragments, and debris after lithotripsy are often left in the kidney due to the lack of effective retrieval tools and limited outflow channels [5]. Most approaches for dust and debris removal involve passive approaches, such as medication induced diuresis, increasing fluid intake and relaxing of the ureter to encourage stone passage. These passive methods yield low efficiencies requiring months to naturally clear, often leading to stone regrowth and additional surgical procedures [6, 7].

Direct in-scope suctioning (DISS) is a novel feature of flexible ureteroscopes that has garnered significant interest in kidney stone treatment. DISS combines traditional flexible ureteroscopy with a dedicated channel for suction, allowing for simultaneous stone fragmentation and active removal of dust and debris during the procedure. Despite its recent emergence, DISS has already demonstrated several advantages over traditional RIRS. Studies have shown that DISS can significantly improve visualization during the stone fragmentation process and lead to a faster stone clearance rate with minimal impact on intrarenal pressure and temperature [8, 9].

The aim of this study was to evaluate the efficiency of the recently introduced 9.2-Fr flexible ureteroscope with a 5.1-Fr DISS, compared to 7.5-Fr ureteroscopes with and without DISS, in suctioning fluid and stone dust at various deflection angles.

## Methods

**Fig. 1:**
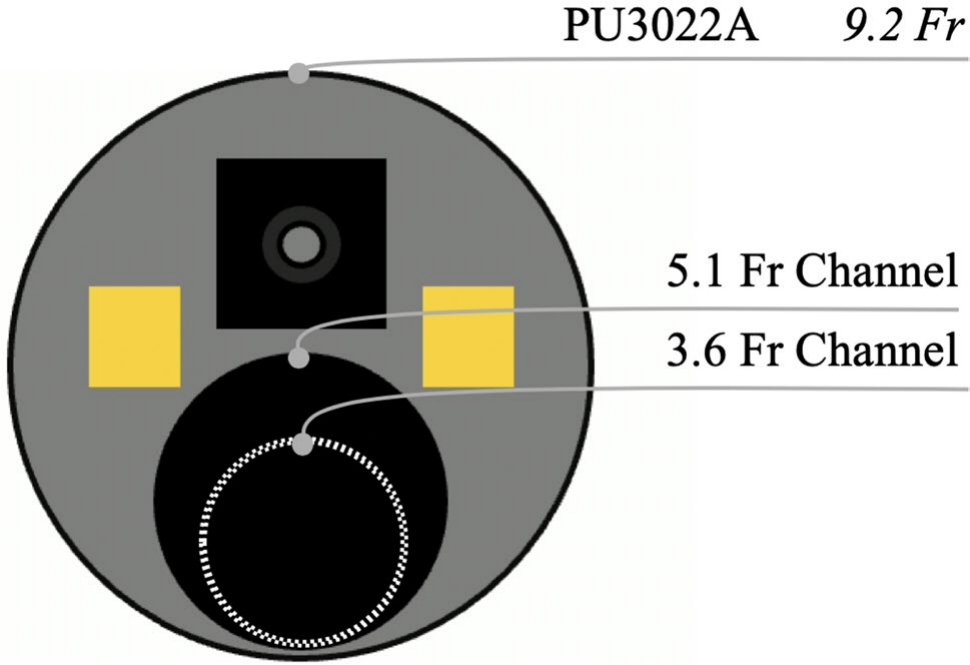
The novel 9.2-Fr with a DISS channel of 5.1-Fr and 7.5-Fr with a DISS channel of 3.6-Fr

**Fig. 2:**
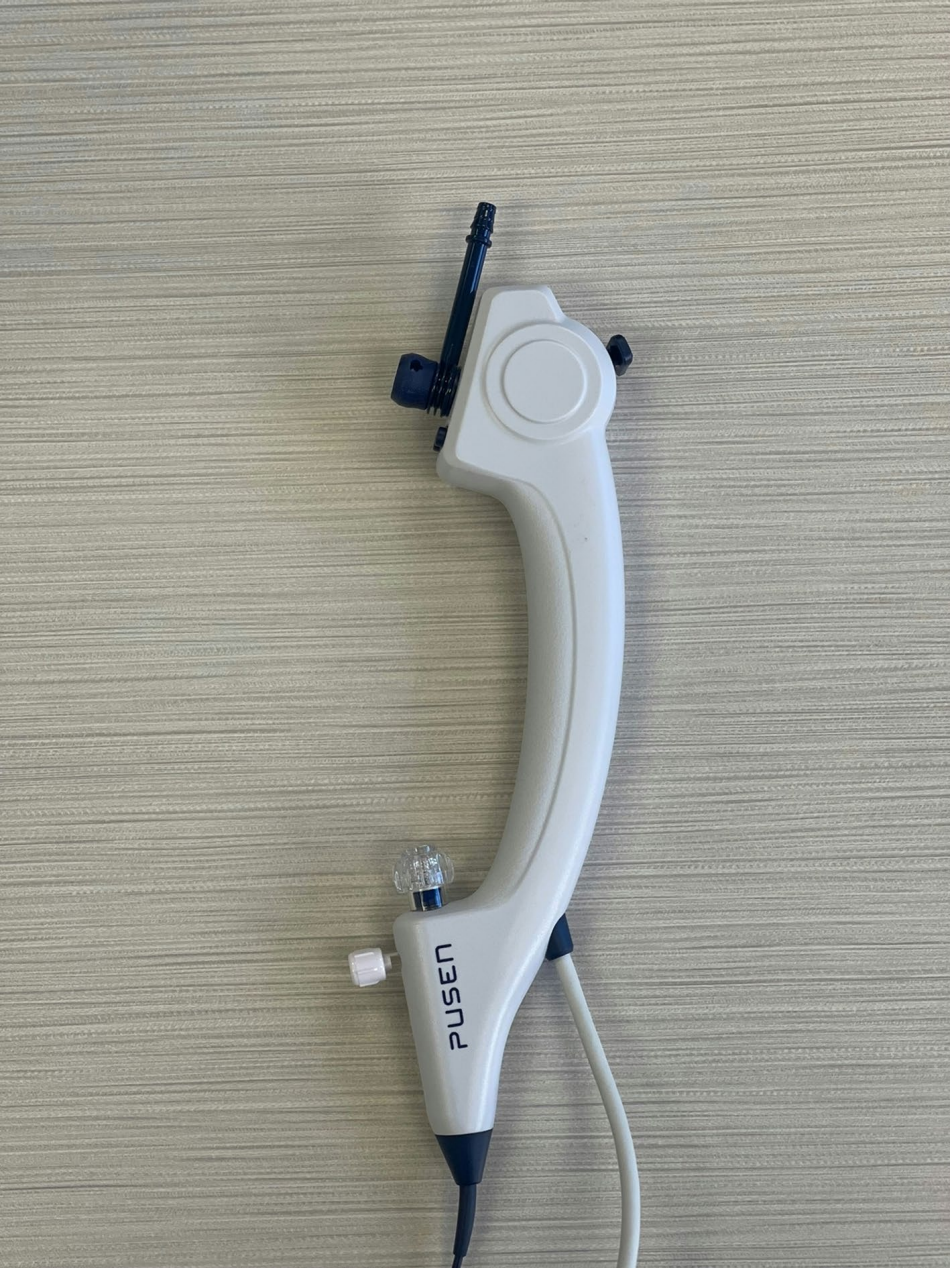
Ergonomic handle of the novel DISS ureteroscope, featuring a DISS button and an integrated suction channel for enhanced procedural efficiency and user comfort

**Ureteroscopes**: Three ureteroscopes were tested in this study including the novel 9.2-Fr with a DISS channel of 5.1-Fr (DISS-U 5.1), 7.5-Fr with a DISS channel of 3.6-Fr (DISS-U 3.6), (Fig. 1), and conventional 7.5-Fr ureteroscope with a regular working/irrigation channel of 3.6-Fr (CU 3.6), (Pusen Medical, Zhuhai, China). The DISS ureteroscopes feature a handle with an integrated suction port and a suction button designed to improve surgeon control over irrigation and suction. Compared to the conventional ureteroscope which the irrigation channel is at 150° angle, the new DISS ureteroscopes have a straight working channel intended to facilitate instrument passage and improve irrigation and aspiration (Fig. 2).

**Fluid suctioning efficiency**: To assess the efficiency in suctioning fluid, we measured the time required to suction 50-mL of saline for all three ureteroscopes and calculated the corresponding suction rate (ml/min). The ureteroscopes with DISS channels were connected to the Neptune 3 Waste Management System (Stryker, Portage, MI) set at maximum suction setting. For the conventional ureteroscope, a 60-mL Luer lock syringe was connected to the working channel and manually suctioned. The fluid was suctioned from a beaker, and the ureteroscope angles were adjusted to 0°, 90° or 140° to simulate real-time ureteroscopy in the upper, interpolar and lower pole of the kidney. The ureteroscope was held by the surgeon at a 90° elbow flexion to simulate an ergonomic position used clinically during flexible ureteroscopy. Irrigation efficiency was tested with an empty channel, a 2.2-Fr NCircle^®^ Nitinol Tipless Stone Extractor (Cook Medical, Bloomington, IN), and a 0.038 Glidewire^®^ Hydrophilic Coated guidewire (Terumo Medical, Tokyo, Japan). This procedure was repeated five times for each trial.

**Fragment evacuation efficiency**: Phantom Bego stones with Calcium Oxalate Monohydrate (COM) urolith density were created in our laboratory and hammered into small fragments that were then passed sequentially through 0.25-, 0.5-, and 1-mm metal sieves (Labalpha, Minneapolis, MI) into collection containers. This created 3 groups of stone fragments between 0.1 and 0.25, 0.1–0.5 and 0.1–1 mm. One-gram dry masses were obtained for each sample using a digital scale to make the samples consistent and equal among the groups.

Fragment evacuation efficiency was quantified as the time recorded from the start of suctioning until the complete evacuation of the 1 g of fragments placed into a receptacle with continuous saline irrigation. Time for removal of all stone fragments was recorded in seconds and clearance rate was calculated in g/min. Stone dust and particles were suctioned without agitation to mimic the actual clinical environment where stone particles settle in a calyx. In the groups with larger stone particles, a maximum suctioning time of 5 min was allowed before stopping the experiment. Complete stone removal was established by the surgeon and confirmed by two independent reviewers.

After each trial, the ureteroscope was flushed with 10 mL of normal saline and a guidewire was passed to dislodge any fragments remaining in the working channel of the ureteroscope. After each trial the kidney beaker was flushed to ensure that no visible fragments remained in the beaker.

**Statistical analysis**: All experiments were performed in quintuplicate (*n* = 5) and results are presented as mean ± SD. Data normality was assessed with the Shapiro-Wilk test. Group comparisons were conducted using one-way ANOVA with Tukey’s post-hoc test or the Kruskal-Wallis test with Dunn’s correction, depending on data distribution. Pairwise comparisons used two-tailed t-tests or Mann-Whitney U tests. Linear regression assessed the relationship between evacuation time and fragment mass. A p-value < 0.05 was considered significant. Analyses were performed using Jamovi version 3.0 (The Jamovi Project, www.jamovi.org).

## **Results**

### Fluid suctioning

Both DISS-U had significantly higher suction rates than the CU 3.6 with an empty channel, or with either a guidewire, or a 2.2 Fr NCircle^®^ in the working channel, at all measured angles (*p* < 0.001 for all; Table 1). The DISS-U 5.1 had the highest suction rate in all measured categories (*p* < 0.001 for all; Table 1). When suctioning with the CU 3.6 at 0°, the use of a 2.2-Fr basket decreased the suction rate by 71.7% (*p* < 0.001), and the use of a 0.038 guidewire decreased the flow rate by 93.9% (*p* < 0.001). With the DISS-U 3.6 at 0°, the use of a 2.2-Fr basket decreased the suction rate by 66.0% (*p* < 0.001). The use of a 0.038 guidewire decreased the flow rate by 95.8% (*p* < 0.001). With the 5.1-Fr DISS-U at 0°, the use of a 2.2-Fr basket decreased the suction rate by 28.6% (*p* < 0.001), with the use of a 0.038 guidewire decreasing the flow rate by 51.3% (*p* < 0.001).

**Table 1:** Suction rate ± standard deviation (ml/min) among the three ureteroscopes with an empty channel, and with a 0.038 guidewire, and 2.2 Fr basket in the working channel

	CU 3.6 Fr	DISS-U 3.6 Fr	DISS-U 5.1 Fr	*p*-value
Empty Channel				
0°	81.2 ± 5.23	136 ± 8.12	273 ± 14.4	< 0.001
90°	78.1 ± 6.72	121 ± 2.81	306 ± 8.64	< 0.001
140°	77.2 ± 7.07	113 ± 2.07	262 ± 17.9	< 0.001
**0.038 Glidewire**				
0°	4.95 ± 0.537	5.67 ± 0.154	133 ± 4.16	< 0.001
90°	4.66 ± 0.275	5.83 ± 0.125	126 ± 3.73	< 0.001
140°	4.58 ± 0.272	5.57 ± 0.211	112 ± 4.74	< 0.001
**2.2Fr NCircle basket**				
0°	23.0 ± 1.81	46.2 ± 3.43	195 ± 6.83	< 0.001
90°	21.3 ± 2.89	46.2 ± 2.20	184 ± 9.03	< 0.001
140°	21.9 ± 1.33	39.8 ± 6.46	173 ± 9.87	< 0.001

Bold values indicates the size of stone particles used for that set of measurements.

CU 3.6 Fr—conventional 7.5-Fr ureteroscope with 3.6-Fr working channel

DISS-U 3.6 Fr—7.5-Fr ureteroscope with 3.6-Fr DISS channel

DISS-U 5.1 Fr—9.2-Fr ureteroscope with 5.2-Fr DISS channel

### Dust evacuation

Both DISS-U had significantly higher suction rates in evacuating 1 g of 0.1 to 0.25 mm stone dust compared to the CU 3.6 at 0°, 90°, and 140° (*p* < 0.001 for all; Table 2). Of the ureteroscopes, the DISS-U 5.1 had the highest suction rate in evacuating 1 g of 0.1–0.25 mm stone dust (*p* < 0.001 for all; Table 2). Neither the CU 3.6 nor the DISS-U 3.6 were able to evacuate 0.5 mm stone particles. The 5.1-Fr DISS-U evacuated 1 g of 0.1–0.5 mm stone significantly faster from the upper pole (0.578 ± 0.19 g/min) compared to the lower pole (0.163 ± 0.031 g/min; *p* < 0.001). The 5.1 DISS-U also evacuated 1 g of 0.5 mm stone at a significantly faster rate in the interpole (0.480 ± 0.096 g/min) than the lower pole (*p* = 0.004). There was no significant difference between the upper and interpolar region (*p* = 0.453).

**Table 2:** Suction rate ± standard deviation (g/min) of stone dust among the three ureteroscopes with an empty channel at different angles and different stone sizes

	CU 3.6 Fr	DISS-U 3.6 Fr	DISS-U 5.1 Fr	*p*-value
**0.25 mm**				
0°	0.463 ± 0.254	2.52 ± 0.583	4.29 ± 0.388	< 0.001
90°	0.287 ± 0.0713	1.84 ± 0.720	4.16 ± 0.419	< 0.001
140°	0.182 ± 0.0296	0.63 ± 0.145	3.92 ± 0.508	< 0.001
**0.5 mm**				
0°	n/a	n/a	0.578 ± 0.19	n/a
90°	n/a	n/a	0.480 ± 0.096	n/a
140°	n/a	n/a	0.163 ± 0.031	n/a

Bold values indicates the specific instruments placed in the working channel during measurement.

CU 3.6 Fr—conventional 7.5-Fr ureteroscope with 3.6-Fr working channel

DISS-U 3.6 Fr—7.5-Fr ureteroscope with 3.6-Fr DISS channel

DISS-U 5.1 Fr—9.2-Fr ureteroscope with 5.2-Fr DISS channel

## Discussion

The DISS-U 5.1 had the shortest time to evacuate 50 mL of saline and the fastest rate of fragment evacuation at all angles compared to the other ureteroscopes. The DISS-U 3.6 was significantly slower than the DISS-U 5.1 but faster than the CU 3.6 in both fluid and fragment evacuation. The largest fragment that could be removed through the CU 3.6 and DISS-U 3.6 was 0.25 mm, while the largest fragment that could be removed through the DISS-U 5.1 was 0.5 mm. None of the scopes could suction stone fragments 1 mm in size without plugging.

With the rising global prevalence of urolithiasis, ureteroscopy has become the most widely adopted procedure for managing renal and ureteral stones [1]. It is also one of the fastest-evolving areas in urologic technology, with recent advancements including thulium fiber lasers, various ancillary instruments, and single-use ureteroscopes offering improved visualization and deflection. However, despite these technological innovations, stone-free rates remain relatively low, ranging from 55 to 70% when assessed using postoperative CT [7, 10]. Even more concerning are the high recurrence rates of kidney stones after surgical intervention, with studies showing recurrence rates of 50% following URS [7, 10].

Traditionally, it was believed that small fragments and dust left behind after RIRS could be safely passed by patients within a few weeks of the procedure. However, a study by Kang and colleagues showed that more than half of the remnants can persist in the kidney for up to 2 years [6]. In their study, up to 40% of patients experienced stone growth after 2 years. Similarly, in a study by Chew and colleagues, 59% of patients experienced stone growth and nearly half (44%) of patients with residual stone fragments after RIRS required further interventions [7]. These recent studies suggest that even small fragments can lead to stone regrowth and recurrence, highlighting the importance of absolute stone-free status.

DISS technology for ureteroscopy has recently emerged as an area of interest in the field of endourology. In a survey conducted by Salka and Ghani on the practice patterns and preferences for next-generation flexible ureteroscopes, over 90% of respondents considered fragment and fluid suctioning to be highly desirable features [11]. Recently, Schneider et al. conducted the first in vitro study evaluating DISS, examining stone fragment clearance using syringe aspiration for fragments smaller than 1 mm and 0.5 mm [12]. Despite comparable amounts of dust (< 1 g) in both size categories, complete stone clearance was not achieved, likely due to the heterogeneous composition of larger stone fragments. Additionally, the continuous agitation of stone particles in their study may have interfered with targeted stone aspiration. Nonetheless, their findings indicated that DISS effectiveness largely depends on dust particle size, which is consistent with our study findings. Similar to the 0.25 mm threshold for the CU 3.6 in our study, Keller et al. adopted a 0.25 mm threshold for most stone types and helped establish a clear definition of stone dust [13]. Additionally, a case series described a DISS technique using a suction-enabled 16 Fr cystoscope with a 7 Fr working channel in three female patients allowing the removal of 97% of stone volume [14]. In all three cases, the ureter was pre-dilated to 16 Fr to allow the introduction of the cystoscope into the collecting system. While this technique was highly effective in removing stone fragments, the need to dilate the ureter to 16 Fr posed a notable risk of ureteric injury. The use of a larger working channel likely provides a significant advantage in such procedures.

Madden and colleagues conducted a bench-top study evaluating the performance of a 7.5-Fr ureteroscope with a 3.6-Fr DISS channel for clearing stone particles up to 1–2 mm in size [15]. Similar to our study, they showed that the DISS ureteroscope successfully cleared 100% of dust particles up to 0.25 mm, and the clearance rate was significantly faster compared to manual aspiration for particles between 0.125 and 0.250 mm. However, the study also highlighted the limitations of the system when faced with larger particles, where the clearance rate dropped to 0.09 g/min for heterogeneous mixtures with particles above 0.25 mm. Furthermore, complete clearance of particles larger than 0.25 mm within 180 s could not be achieved, and blockages were encountered during the process. Nedbal and colleagues performed the first prospective, multicenter clinical evaluation of the 7.5 Fr ureteroscope with 3.6-Fr DISS [9]. The study involved a total of 57 patients, with the goal of evaluating the device’s maneuverability, suction efficiency, visibility, and overall clinical outcomes. In their study, participating surgeons consistently rated the DISS feature highly, with 95% finding it helpful in improving visibility and clearing debris during the procedure. This improvement in intraoperative visualization is significant, as it reduced the common “snow globe” effect, where dust from fragmented stones obscures the surgeon’s view, lengthening the procedure. Furthermore, surgeons gave it high ratings for scope placement, visual quality, deflection, and maneuverability, with an overall satisfaction score of 4.1 out of 5. Stone-free rates (SFR) were 84.21%, confirmed through follow-up CT imaging. The study noted that smaller fragments were effectively cleared using suction, allowing for faster procedures and less reliance on endoscopic baskets. However, some surgeons reported that larger fragments were more difficult to aspirate through the 3.6 Fr working channel, indicating a potential limitation in the current design.

Our study builds on these findings and is the first to test the DISS-U 5.1 with its larger working channel designed to overcome the limitations of the prior iteration. One of the novel advantages of the DISS-U 5.1 is its capability to irrigate and suction simultaneously while accommodating either the basket or guidewire in the working channel. Additional benefits of DISS ureteroscopes may include maintaining intrarenal pressure and enhancing intraoperative visualization by clearing debris and dust particles. While suction can aid in stone particle removal, it may also cause abrupt fluctuations in intrarenal pressure due to alternating suction and irrigation, potentially resulting in suboptimal pressures. These fluctuations can lead to complications such as bleeding, venous backflow, sepsis, and mucosal damage, which may cause long-term issues like strictures. Clinical studies are needed to assess the effect of DISS technology upon intrarenal pressure changes and postoperative outcomes.

Our study is the first to demonstrate that all devices suction less efficiently in the lower pole when the ureteroscope is deflected. In comparing the efficiency of 0.25 mm particle evacuation at 140° (lower pole) versus 0° (upper pole), we observed a reduction of 75% for CU 3.6 Fr, 60.69% for DISS-U 3.6 Fr, and 8.62% for DISS-U 5.1 Fr. These findings suggest that the dust evacuation efficiency at greater deflection angles significantly varies across devices, with the CU 3.6 Fr demonstrating the highest reduction and with the DISS-U 5.1 Fr maintaining the most stability in performance. Additionally, the 0.5 mm particle evacuation with DISS-U 5.1 Fr demonstrated a reduction of 71.80% when comparing performance at 140° versus 0°. This is an important finding because the lower pole fragments are most likely to persist resulting in stone growth and recurrence.

Our study is the first to evaluate the 9.2-Fr ureteroscope with 5.1-Fr DISS channel, demonstrating its enhanced suction and dust evacuation capabilities. In an era where ureteroscope technology is trending towards smaller sizes, concerns may arise about the outer diameter of the 9.2-Fr ureteroscope. However, this ureteroscope presents a favorable risk-benefit profile, particularly for treating larger stones when percutaneous nephrolithotomy is contraindicated offering single or staged RIRS. This DISS-U is the same size or smaller than many available flexible videoendoscopes currently on the market. Similarly, the CVAC system, with its 11.9-Fr outer diameter, offers a 7.5-Fr (2.5 mm) DISS channel [16, 17]. Stern and colleagues reported RIRS experience using the CVAC system in 43 patients who were poor surgical candidates for PCNL and only 9.5% of those who anticipated having staged multiple procedures required a second procedure. We believe this represents a significant technological advancement in RIRS, with the potential to improve SFR outcomes in patients undergoing RIRS.

There are some limitations to our study. The bench-top model does not fully replicate the clinical conditions encountered in real patients. While we used Bego stones, they may not perfectly mimic actual kidney stones in terms of their frictional properties and suctioning characteristics. However, this setup provides standardized testing conditions, providing the ability to test stones of known size to identify the maximal suctioning size threshold for different ureteroscopes, providing an objective evaluation of this new technology. Suction was performed in a bench top model, not in an actual kidney which may not completely replicate the fluid dynamics associated with the actual kidney. Suction employed in our model was through a dedicated suction device. We suspect that other suction devices including wall mount suction would provide adequate suction for ureteroscopy. This was not specifically tested in our model. Finally, only one type of basket and guidewire were tested.

## Conclusions

This study is the first to evaluate the novel 9.2-Fr ureteroscope with 5.1-Fr DISS, highlighting its ability to evacuate fragments up to 0.5 mm in size. Both DISS ureteroscopes demonstrated markedly enhanced suctioning capabilities and dust evacuation efficiency compared to conventional ureteroscopes. Clinical studies are needed to elucidate the role of DISS in ureteroscopy.

## Data Availability

No datasets were generated or analysed during the current study.

## Abbreviations

RIRS: Retrograde intrarenal surgery
DISS: Direct-in-scope suction
URS: Ureteroscopy
fURS: Flexible ureteroscopy
